# Prediction of Hepatocellular Carcinoma After Direct‐Acting Antiviral Therapy Using Agile 3+ and End‐of‐Treatment Alpha‐Fetoprotein Levels in Patients With Hepatitis C

**DOI:** 10.1002/jgh3.70296

**Published:** 2025-10-22

**Authors:** Akifumi Kuwano, Masayoshi Yada, Kosuke Tanaka, Taikan Hamomoto, Kazuki Kurosaka, Hideo Suzuki, Kenta Motomura

**Affiliations:** ^1^ Department of Hepatology Aso Iizuka Hospital Fukuoka Japan; ^2^ Department of Gastroenterology Ichinomiyanishi Hospital Ichinomiya Aichi Japan

**Keywords:** Agile 3+ score, direct‐acting antivirals, hepatitis C virus, hepatocellular carcinoma, α‐fetoprotein

## Abstract

Direct‐acting antivirals (DAAs) have dramatically improved sustained virological response (SVR) rates in patients with hepatitis C virus (HCV) infection. However, the risk of hepatocellular carcinoma (HCC) remains even after achieving SVR. We previously reported that alpha‐fetoprotein (AFP) levels at the end of treatment (EOT) were associated with the occurrence of HCC after achieving sustained virologic response (SVR). Here, to improve predictive accuracy by incorporating the Agile 3+ score among 502 patients who received DAA therapy for HCV infection between September 2017 and July 2024, we excluded those who developed HCC within 1 year of treatment and included 337 patients with chronic hepatitis or compensated cirrhosis, who had no prior HCC and underwent transient elastography before treatment. A scoring system was developed by assigning 1 point for Agile 3+ score ≥ 0.9398 and 1 point for EOT‐AFP ≥ 3.8 ng/mL. The cumulative HCC incidence was analyzed in relation to the total score. Cox proportional hazards models were used to assess independent risk factors. During follow‐up, 15 patients (4.5%) developed HCC. Patients with a score of ≥ 1 had a significantly higher HCC risk (*p* < 0.001). Agile 3‐AFP score ≥ 1 (hazard ratio (HR) 12.65, 95% CI 1.38–115.49, *p* = 0.02) and GGT (HR 1.00, 95% CI 1.00–1.01, *p* = 0.02) remained independent predictors of HCC occurrence. A simple scoring system combining the pretreatment Agile 3+ score and EOT‐AFP levels may be useful for long‐term risk stratification of HCC after DAA therapy in HCV‐infected patients.

**Abbreviations**
AFP: α‐fetoprotein
Alb: albumin
ALD: alcohol‐related liver disease
ALT: alanine aminotransferase
aMAP: age‐male‐albumin‐bilirubin–platelets
AST: aspartate aminotransferase
AUC: area under the curve
BMI: body mass index
BR + GZR: elbasvir and grazoprevir
CAP: controlled attenuation parameter
CI: confidence interval
C‐index: concordance index
DAAs: direct‐acting antivirals
DM: diabetes mellitus
EOT: end of treatment
FIB‐4 index: fibrosis‐4 index
GGT: γ‐glutamyl transferase
GLE/PIB: glecaprevir and pibrentasvir
HCC: hepatocellular carcinoma
HCV: hepatitis C virus
HDL: high‐density lipoprotein
HR: hazard ratio
IFN: interferon
LC: liver cirrhosis
LSM: liver stiffness measurement
MASLD: metabolic dysfunction‐associated steatotic liver disease
Plt: platelet count
QR: interquartile range
ROC: receiver operating characteristic
SOF/LDV: sofosbuvir and ledipasvir
SOF + RBV: sofosbuvir and ribavirin
SOT: start of treatment
SVR: sustained virological response
TB: total bilirubin
TC: total cholesterol
TE: transient elastography
TG: triglyceride
WBC: white blood cell

Hepatitis C virus (HCV) infection remains a major global health concern and a leading cause of hepatocellular carcinoma (HCC) [1]. The advent of direct‐acting antiviral agents (DAAs) has revolutionized HCV treatment, achieving sustained virological response (SVR) rates exceeding 95% in most patient populations [2]. However, despite successful viral eradication, the risk of hepatocarcinogenesis persists [3]. A multicenter study reported a 3‐year cumulative incidence of HCC of 4.9% following DAA therapy [4]. Several recent studies have identified risk factors for HCC development after SVR, including elevated serum α‐fetoprotein (AFP) levels, low platelet count, hypoalbuminemia, older age, and a higher fibrosis‐4 (FIB‐4) index [5, 6, 7, 8, 9].

Sano et al. further reported that metabolic dysfunction‐associated steatotic liver disease (MASLD) is a significant risk factor for HCC in elderly patients with HCV eradication [10]. Additionally, obesity and heavy alcohol consumption have been associated with increased post‐SVR HCC risk [11].

The Agile 3+ score is a composite noninvasive test designed to detect advanced fibrosis in patients with MASLD or alcohol‐related liver disease (ALD) [12, 13]. This score integrates liver stiffness measurement (LSM) via transient elastography (TE) with routine laboratory and clinical parameters, including platelet count, aspartate aminotransferase (AST), alanine aminotransferase (ALT), age, sex, and diabetes mellitus (DM) status, to generate a continuous risk score ranging from 0 to 1. In patients with metabolic MASLD, the Agile 3+ score demonstrated superior diagnostic performance over FIB‐4 (AUC 0.86 vs. 0.79). Similarly, in ALD, the Agile 3+ score outperformed FIB‐4 (AUC 0.92 vs. 0.80) [12]. These findings indicate that the Agile 3+ score more accurately distinguishes patients with and without advanced fibrosis, making it a more precise noninvasive diagnostic tool than FIB‐4 in both MASLD and ALD populations. While its utility in fibrosis staging is well established, the prognostic value of the Agile 3+ score for predicting HCC after HCV eradication has remained unclear.

Serum AFP levels at the end of treatment (EOT) have also been reported as predictive markers for HCC risk after SVR [14, 15]. In our previous study, we demonstrated that an EOT‐AFP level greater than 3.8 ng/mL was significantly associated with increased HCC incidence following treatment [16]. However, LSM, which reflects the severity of hepatic fibrosis, was not included in that analysis.

In the present study, we aimed to evaluate the association between the Agile 3+ score and long‐term HCC risk following DAA‐induced SVR. Furthermore, we developed a newly described scoring model combining the Agile 3+ score with EOT‐AFP levels to enhance HCC risk stratification in patients with chronic hepatitis C who achieved SVR.

## 1 Methods

### 1.1 Study Design and Population

This retrospective cohort study was conducted at Aso Iizuka Hospital. The present study was conducted following the guidelines of the Declaration of Helsinki and was approved by the Ethics Committee of Aso Iizuka Hospital (approval No. 17115). Written informed consent for participation in the study was obtained from all patients prior to DAA treatment. We reviewed the medical records of 502 patients with chronic hepatitis C who received DAA therapy between September 2017 and September 2024. Patients were included if they met the following criteria: (1) achieved SVR, (2) had no prior history of HCC before initiating DAA therapy, (3) underwent TE evaluation before treatment, and (4) had either chronic hepatitis or compensated cirrhosis. Exclusion criteria were: (1) coinfection with hepatitis B virus or human immunodeficiency virus, or the presence of other liver diseases such as primary biliary cholangitis or autoimmune hepatitis; (2) prior history of HCC or failure to achieve SVR12 after DAA treatment; (3) decompensated cirrhosis; and (4) HCC occurrence within 1 year after the EOT. LC was diagnosed based on liver histology or transient elastography or the presence of gastro‐esophageal varices. After applying these criteria, a total of 337 patients were eligible for analysis. Follow‐up started on the date of EOT. Patients were followed until the earliest of HCC, death, liver transplantation, and loss to follow‐up. Non‐HCC cases were censored at the last HCC‐free date.

### 1.2 MASLD and ALD

MASLD was defined as the presence of hepatic steatosis (controlled attenuation parameter (CAP) ≥ 288 dB) in combination with at least one metabolic risk factor. ALD was defined as steatosis plus an average daily alcohol intake exceeding 50 g for women or 60 g for men, together with at least one metabolic risk factor. Metabolic risk factors were determined based on the updated consensus criteria and included: systolic/diastolic blood pressure ≥ 130/85 mmHg or current use of antihypertensive medication; body mass index (BMI) ≥ 23 kg/m^2^ or waist circumference > 94 cm in men or > 80 cm in women; fasting plasma glucose ≥ 100 mg/dL, hemoglobin A1_c_ ≥ 5.7%, or use of antidiabetic medication; serum triglyceride level ≥ 150 mg/dL or use of lipid‐lowering agents; and high‐density lipoprotein (HDL) cholesterol level < 40 mg/dL in men or < 50 mg/dL in women, or use of lipid‐lowering agents [17].

### 1.3 DAA Treatment

Of the 338 patients, 24 were treated with sofosbuvir and ribavirin for 12 weeks; 40 were treated with sofosbuvir and ledipasvir for 12 weeks; 28 received elbasvir and grazoprevir for 12 weeks; and the remaining 246 patients were treated with glecaprevir and pibrentasvir for 8 to 12 weeks (Table 1).

**TABLE 1:** Baseline characteristics of patients.

Characteristic	*n* (%) or $\tilde{x}$ (IQR)
*n*	337
Age (years)	62 (53–72)
Age > 70 years	110 (32.6%)
Male/female	195/142
BMI	22.8 (20.5–25.6)
LC	66 (19.6%)
MASLD	58 (17.2%)
ALD	52 (15.4%)
DM	60 (17.8%)
Treatment	
SOF + RBV	24 (7.1%)
SOF/LDV	40 (12.0%)
EBR + GZR	28 (8.3%)
GLE/PIB	245 (72.6%)
Genotype 1B/2A/2B	145/125/67
HCV RNA (log IU/mL)	6.2 (3.4–6.9)
CAP	218.0 (189.8–259.0)
LSM	6.6 (4.8–10.7)
Blood test at SOT	
Plt (×10^4^/mm^3^)	17.8 (13.6–23.4)
TB (mg/dL)	0.7 (0.6–1.0)
AST (IU/L)	41.0 (25.0–73.3)
ALT (IU/L)	39.0 (22.0–83.3)
Alb (g/dL)	4.0 (3.8–4.3)
GGT (IU/L)	38.0 (20.8–89.3)
TC (mg/dL)	169.0 (147.0–193.0)
TG (mg/dL)	100.0 (74.0–135.0)
AFP (ng/mL)	3.5 (2.3–6.7)
FIB‐4 index	2.48 (1.39–3.91)
Agile 3+ score	0.760 (0.513–0.925)
Blood test at EOT	
Plt (×10^4^/mm^3^)	19.3 (14.8–23.5)
TB (mg/dL)	0.9 (0.6–1.2)
AST (IU/L)	20.0 (17.0–28.0)
ALT (IU/L)	15.0 (11.0–23.0)
Alb (g/dL)	4.1 (3.9–4.3)
GGT (IU/L)	20.0 (14.0–29.5)
TC (mg/dL)	178.5 (152.0–200.0)
TG (mg/dL)	98.5 (70.0–140.0)
AFP (ng/mL)	2.9 (1.9–4.7)
FIB‐4 index	1.83 (1.23–2.77)
aMAP scores	68.1 (61.9–72.9)
Time from EOT (years)	3.97 (2.05–5.78)

*Note:* Data are expressed as median ($\tilde{x}$) and interquartile range (IQR) or frequency (percent).

Abbreviations: AFP, α‐fetoprotein; Alb, albumin; ALD, alcohol‐related liver disease; ALT, alanine aminotransferase; aMAP, age‐male‐albumin‐bilirubin–platelets; AST, aspartate aminotransferase; BMI, body mass index; CAP, controlled attenuation parameter; DM, diabetes mellitus; EBR + GZR, elbasvir and grazoprevir; EOT, end of treatment; FIB‐4 index, fibrosis‐4 index; GGT, γ‐glutamyl transferase; GLE/PIB, glecaprevir and pibrentasvir; HCV, hepatitis C virus; IQR, interquartile range; LC, liver cirrhosis; LSM, liver stiffness measurement; MASLD, metabolic dysfunction associated steatotic liver disease; Plt, platelet count; SOF/LDV, sofosbuvir and ledipasvir; SOF + RBV, sofosbuvir and ribavirin; SOT, start of treatment; TB, total bilirubin; TC, total cholesterol; TG, triglyceride; $\tilde{x}$, median.

### 1.4 Follow‐Up and Outcomes

The primary endpoint was the development of HCC more than 1 year after achieving SVR. HCC surveillance was conducted every 3 to 6 months following DAA treatment, using serum AFP measurements and imaging modalities such as ultrasonography, CT, and magnetic resonance imaging. The median duration of follow‐up was 3.97 years, with an interquartile range (IQR) of 2.05 to 5.78 years.

### 1.5 HCV RNA

HCV RNA was isolated from 200 μL of serum using the miRNeasy Serum/Plasma Kit (Qiagen, Hilden, Germany) following the manufacturer's protocol. Viral RNA levels were quantified utilizing a commercially available TaqMan‐based real‐time PCR assay (COBAS AmpliPrep/COBAS TaqMan HCV Test, Version 2; Roche Molecular Diagnostics, Pleasanton, CA, USA), which has a lower limit of quantification of 1.6 log_10_ IU/mL and a detection threshold of 1.2 log_10_ IU/mL, as specified by the manufacturer. Genotyping of HCV was carried out using the Versant HCV Genotype 2.0 Line Probe Assay (LiPA 2.0; Siemens Healthineers, Erlangen, Germany). Additionally, viral sequencing was employed to differentiate between genotypes 1a, 1b, 2, 3, 4, and 6, in line with the manufacturer's instructions.

SVR was defined as undetectable serum HCV RNA levels, measured at least 12 weeks after completion of therapy and below the lower detection limit of the assay [18].

### 1.6 FIB‐4 Index

The FIB‐4 index was used to clinically assess the degree of liver fibrosis both before and after DAA therapy. It was calculated using the following formula: FIB‐4 index = [AST (IU/L) × age (years)]/[platelet count (10^9^/L) × √ALT (IU/L)] [19].

### 1.7 Agile 3+ Score

The Agile 3+ score comprises six clinical and laboratory factors: LSM (kPa), age, sex, DM status, AST, and platelet count [20]. The score is scaled from 0 to 1 and was developed to enhance the diagnostic accuracy for advanced fibrosis in individuals with MASLD or ALD. TE was conducted using a FibroScan (Echosens, Paris, France) transient elastography measurement device, applying either the M‐ or XL‐probe depending on patient characteristics. CAP and LSM values were obtained through FibroScan. Given the possibility of measurement errors due to subcutaneous fat when using the M‐probe in obese patients, the XL‐probe was preferentially employed in such cases. Patients were examined while they were supine with the right arm maximally abducted to expose the right liver lobe. Measurements were considered valid if there were at least 10 readings, with a ≥ 60% success rate and an interquartile range < 30%. The median of these valid measurements was used for analysis.

### 1.8 Age‐Male‐Albumin–Bilirubin–Platelets (aMAP) Score

aMAP scores were calculated using data for age, gender, serum total bilirubin, serum albumin, and platelet count obtained at a pretreatment examination, as follows: aMAP risk score = ({0.06 × age + 0.89 × gender (male: 1, female: 0) + 0.48 × (log_10_ serum total bilirubin [μmol/L] × 0.66) + (serum albumin [g/L] × −0.085) −0.01 × platelet count (10^3^/mm^3^)} + 7.4)/14.77 × 100 [21].

### 1.9 Statistical Analysis

The data are presented as median [IQR] (Tables 1 and 2). Cumulative incidence of HCC was estimated using a Kaplan–Meier method and compared across risk score groups using a log‐rank test. The prognostic value of the composite score was assessed using univariate and multivariate Cox proportional hazards models. Hazard ratios (HRs) and 95% confidence intervals (CIs) were calculated. Receiver operating characteristic (ROC) curve analysis was used to determine the optimal cutoff point for the Agile 3+ score. We assessed discrimination of two prespecified binary predictors using Harrell's concordance index (C‐index) for right‐censored data with ties counted as 0.5. All *p*‐values were derived from two‐tailed tests, and differences with *p* < 0.05 were considered significant. All statistical analyses were performed using JMP Pro software (version 17; SAS Institute Inc., Cary, NC).

**TABLE 2:** Characteristics of patients with or without HCC after DAA treatment.

Characteristic	HCC occurrence	No HCC occurrence	*p*
*n*	15	322	
Age (years)	63 (55–73)	61.5 (52.8–72.0)	0.58
Age > 70 (years)	6 (40.0%)	104 (32.3%)	0.54
Male/female	12/3	183/139	0.06
BMI	23.9 (22.0–25.3)	22.7 (20.4–25.6)	0.71
LC	10 (66.7%)	56 (17.4%)	< 0.001
MASLD/ALD	9 (60.0%)	101 (31.4%)	0.03
DM	4 (26.7%)	56 (17.4%)	0.38
CAP (dB)	235.0 (190.0–254.0)	218.0 (189.0–259.5)	0.91
LSM (kPa)	14.8 (7.6–35.3)	6.4 (4.8–10.3)	< 0.001
Blood test results at SOT			
Plt (×10^4^/mm^3^)	10.0 (8.1–18.2)	17.9 (13.8–23.7)	< 0.001
TB (mg/dL)	0.9 (0.7–1.3)	0.7 (0.6–1.0)	< 0.001
AST (IU/L)	79.0 (24.0–109.0)	39.5 (25.0–71.3)	0.07
ALT (IU/L)	54.0 (28.0–128.0)	38.5 (21.0–76.0)	0.17
GGT (IU/L)	138.0 (32.0–196.0)	37.0 (20.0–85.3)	0.004
Alb (g/dL)	3.9 (3.6–4.0)	4.1 (3.8–4.3)	0.03
TC (mg/dL)	164.0 (149.0–178.0)	169.0 (146.0–194.0)	0.54
TG (mg/dL)	101.0 (76.0–241.4)	100.0 (74.0–135.0)	0.82
AFP (ng/mL)	6.6 (3.7–19.6)	3.3 (2.3–6.1)	0.53
FIB‐4 index	4.30 (2.84–7.76)	2.40 (1.38–3.80)	< 0.001
Agile 3+ score	0.995 (0.825–0.993)	0.748 (0.505–0.920)	< 0.001
Blood test results at EOT			
Plt (×10^4^/mm^3^)	10.4 (7.9–23.5)	19.4 (15.1–23.6)	0.007
TB (mg/dL)	1.15 (0.88–1.55)	0.8 (0.6–1.2)	0.23
AST (IU/L)	24.0 (20.0–44.0)	20.0 (16.5–28.0)	0.003
ALT (IU/L)	16.0 (12.0–45.0)	14.0 (11.0–22.0)	0.008
Alb (g/dL)	3.9 (3.7–4.1)	4.1 (3.9–4.3)	0.25
GGT (IU/L)	44.0 (20.0–111.0)	19.0 (14.0–29.0)	< 0.001
TC (mg/dL)	168.0 (151.8–179.0)	180.0 (152.0–201.0)	0.34
TG (mg/dL)	111.0 (73.0–175.8)	97.0 (69.5–140)	0.44
AFP (ng/mL)	4.4 (3.4–9.2)	2.8 (1.9–4.6)	0.008
FIB‐4 index	2.67 (1.50–5.00)	1.82 (1.20–2.74)	0.001
aMAP scores	72.1 (65.7–77.4)	68.0 (61.5–72.6)	0.01
Time from EOT (years)	3.03 (1.81–4.14)	4.03 (2.07–5.81)	0.15

*Note:* Data are expressed as median ($\tilde{x}$) and interquartile range (IRQ) or frequency (percent).

Abbreviations: AFP, α‐fetoprotein; Alb, albumin; ALD, alcohol‐related liver disease; ALT, alanine aminotransferase; aMAP, age‐male‐albumin‐bilirubin–platelets; AST, aspartate aminotransferase; BMI, body mass index; CAP, controlled attenuation parameter; DM, diabetes mellitus; EOT, end of treatment; FIB‐4 index, fibrosis‐4 index; GGT, γ‐glutamyl transferase; HCC, hepatocellular carcinoma; HCV, hepatitis C virus; IQR, interquartile range; LC, liver cirrhosis; LSM, liver stiffness measurement; MASLD, metabolic dysfunction associated steatotic liver disease; Plt, platelet count; SOT, start of treatment; TB, total bilirubin; TC, total cholesterol; TG, triglyceride; $\tilde{x}$, median.

## 2 Results

### 2.1 Patient Characteristics

The baseline characteristics of the study population are presented in Table 1. A total of 338 patients were enrolled, with a median age of 62 years (IQR, 52–72 years), and 110 individuals (32.6%) were aged over 70. The cohort included 195 men and 142 women. Their median BMI was 22.8 kg/m^2^. Liver cirrhosis was identified in 66 patients (19.6%). Before initiating DAA therapy, the median CAP was 218.5 dB (IQR, 189.8–259.0 dB), and the median LSM was 6.6 kPa (IQR, 4.8–10.7 kPa).

Table 2 displays the post‐DAA treatment characteristics of patients stratified by HCC development: the HCC group (*n* = 15), the no HCC group (*n* = 322). Median age did not differ significantly between groups (63.0 [IQR, 55.0–73.0] vs. 61.5 [IQR, 52.8–72.0]) years (*p* = 0.58), nor did the proportion of patients aged over 70 years (40.0% vs. 32.3%, *p* = 0.54). However, liver cirrhosis was significantly more frequent in the HCC group (66.7% vs. 17.4%, *p* < 0.001). The median CAP, reflecting hepatic steatosis, was similar in both groups (235.0 vs. 218.0 dB, *p* = 0.91). Conversely, LSM, a marker of liver fibrosis, was significantly higher in the HCC group (14.8 vs. 6.35 kPa, *p* < 0.001), indicating more severe fibrosis. Additionally, the HCC group exhibited significantly lower platelet counts (10.0 vs. 17.9 × 10^4^/mm^3^, *p* = 0.001), elevated total bilirubin (0.9 vs. 0.7 mg/dL, *p* < 0.001), and decreased serum albumin levels (3.9 vs. 4.1 g/dL, *p* = 0.03) at the start of treatment (SOT). The FIB‐4 index at SOT was also markedly higher in this group (4.30 vs. 2.40, *p* < 0.001). Agile 3+ score remained significantly elevated (0.9445 vs. 0.748, *p* = 0.006). Similar findings were observed at EOT. The HCC group continued to show lower platelet counts (10.4 vs. 19.4 × 10^4^/mm^3^, *p* = 0.007), higher AST (24.0 vs. 20.0 IU/L, *p* = 0.003) and ALT (16.0 vs. 14.0 IU/L, *p* = 0.008) levels, and increased AFP levels (4.4 vs. 2.8 ng/mL, *p* = 0.008), the FIB‐4 index at EOT (2.67 vs. 1.82, *p* = 0.001) and the aMAP scores (72.1 vs. 68.0, *p* = 0.01). There was no significant difference in median follow‐up duration from the EOT between the two groups (3.0 vs. 4.0 years, *p* = 0.15).

### 2.2 Cumulative Incidence Rate of HCC

**FIGURE 1:**
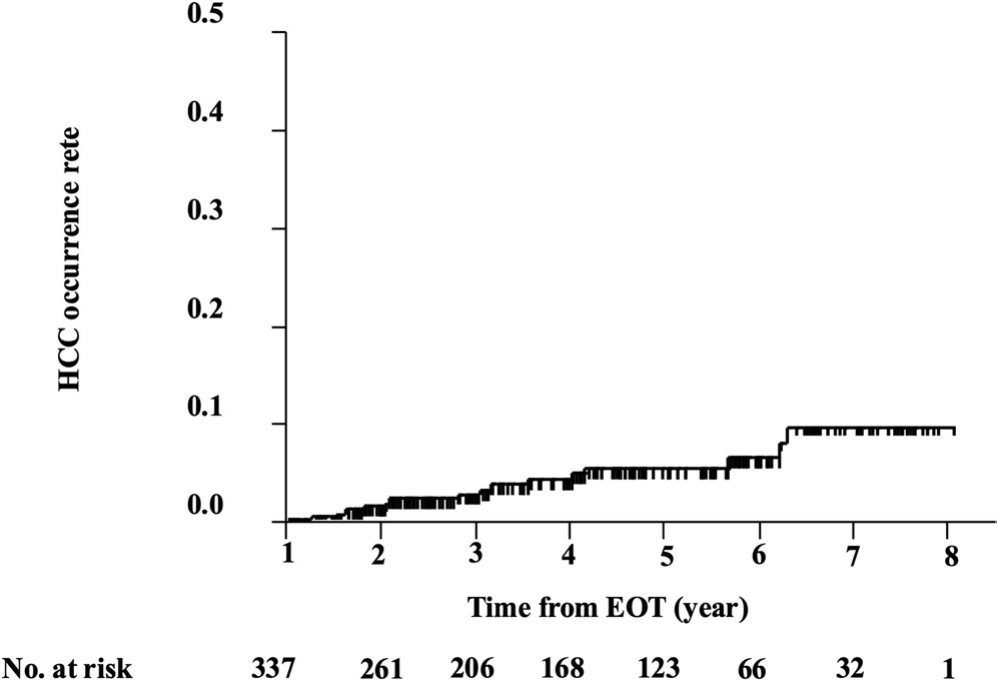
Cumulative incidence of HCC from the end of HCV eradication using DAA treatment. DAA, direct‐acting antiviral; EOT, end of treatment; HCC, hepatocellular carcinoma; HCV, hepatitis C virus.

During the observation period, 15 patients (4.5%) developed HCC more than 1 year after completing DAA therapy. The cumulative incidence of HCC from the EOT was as follows: 1.4% at 2 years, 2.6% at 3 years, 4.7% at 4 years, 5.3% at 5 years, 6.3% at 6 years, and 9.6% at 7 years (Figure 1).

### 2.3 Agile 3+ With AFP Risk Score and HCC Development

The optimal cutoff value for the Agile 3+ score, as determined by ROC curve analysis, was 0.9398 (area under the curve (AUC) 0.75). An Agile 3+ score ≥ 0.9398 was assigned 1 point, and an Agile 3+ score < 0.9398 ng/mL was assigned 0 points. Based on previous studies, a threshold of ≥ 3.8 ng/mL was used to define elevated EOT‐AFP (16). Patients with AFP ≥ 3.8 ng/mL were assigned 1 point, and those with AFP < 3.8 ng/mL were assigned 0 points. The total risk score (Agile 3+ and AFP scores) was calculated as the sum of the Agile 3+ score point and the EOT‐AFP score point, ranging from 0 to 2 (Score 0: *n* = 193 (57.1%), Score 1: *n* = 102 (30.1%), Score 2: *n* = 43 (12.7%)). The combination of EOT‐AFP and the Agile 3+ score demonstrated a stronger association with HCC risk than either factor alone. Specifically, while EOT‐AFP ≥ 3.8 ng/mL and Agile 3+ score ≥ 0.9398 were individually associated with increased risk (HR = 4.11 and 7.40, respectively), the combined score (Agile 3+ and AFP scores ≥ 1) showed an even higher hazard ratio (HR = 19.34, 95% CI 2.54–147.17, *p* = 0.004), suggesting that integrating both parameters may enhance predictive performance (Table 3).

**TABLE 3:** Univariable HRs for HCC occurrence ≥ 1 year following HCV eradication using DAA treatment: EOT‐AFP, Agile 3+ score and their composite scores.

Factor	HR	95% CI	*p*
EOT‐AFP ≥ 3.8 (ng/mL)	4.11	1.40–12.06	0.001
Agile 3+ score ≥ 0.9398	7.40	2.53–21.68	< 0.001
Agile 3+ and AFP scores			
0	Ref		
1	15.47	1.93–123.74	0.01
2	29.12	3.50–242.31	0.002
≥ 1	19.34	2.54–147.17	0.004

*Note:* Data are expressed as hazard ratios (HRs) with 95% confidence intervals (CIs).

Abbreviations: AFP, α‐fetoprotein; CI, confidence interval; DAA, direct‐acting antiviral; EOT, end of treatment; HCC, hepatocellular carcinoma; HCV, hepatitis C virus; HR, hazard ratio.

**FIGURE 2:**
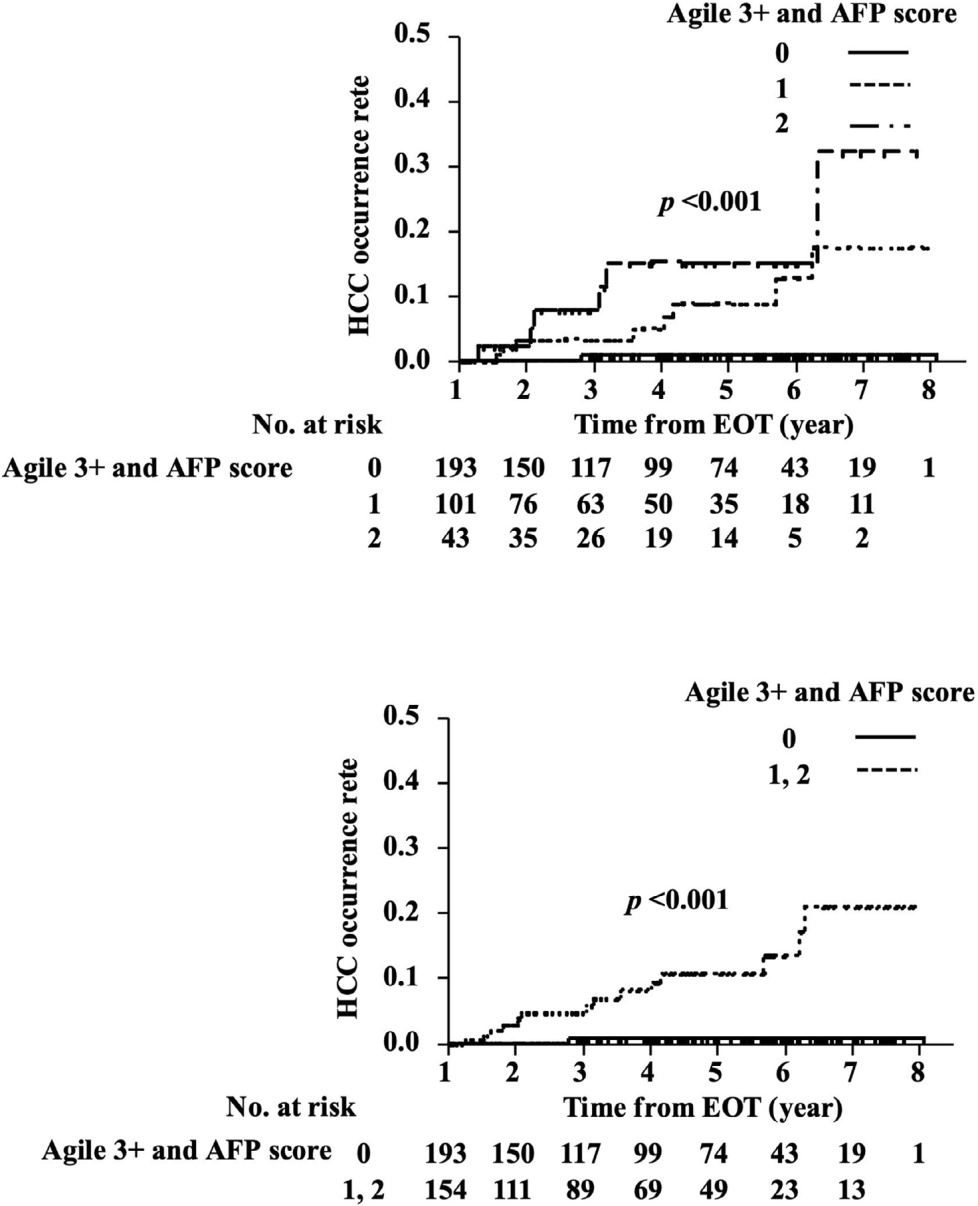
Kaplan–Meier analysis of the cumulative HCC occurrence from the end of HCV eradication, stratified based on Agile 3+ scores (≥ 0.9398: 1 point) and AFP level scores associated with HCC occurrence. AFP, α‐fetoprotein; DAA, direct‐acting antiviral; HCC, hepatocellular carcinoma; HCV, hepatitis C virus.

**FIGURE 3:**
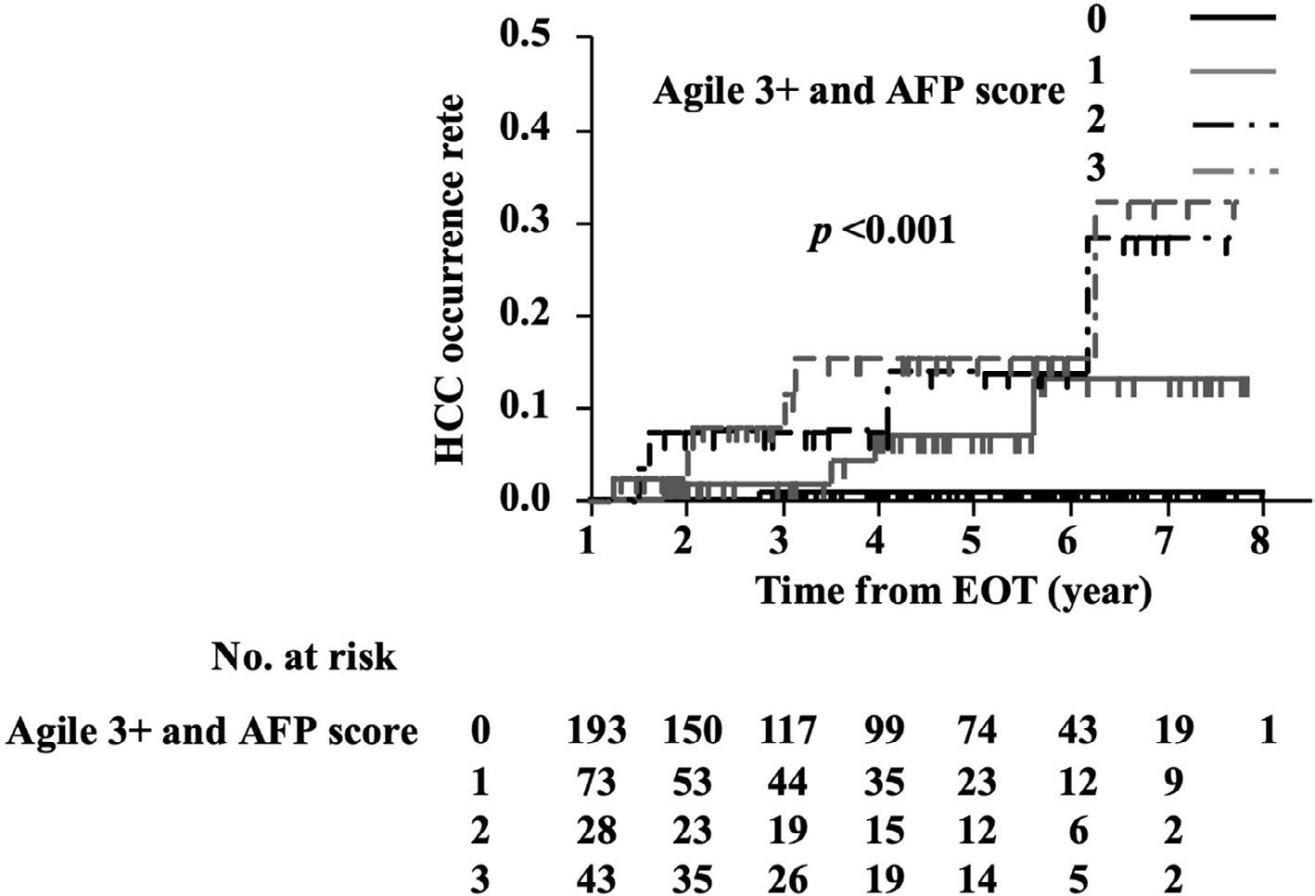
Kaplan–Meier analysis of the cumulative HCC occurrence from the end of HCV eradication, stratified based on Agile 3+ scores (≥ 0.9398: 2 points) and AFP level scores associated with HCC occurrence. AFP, α‐fetoprotein; DAA, direct‐acting antiviral; HCC, hepatocellular carcinoma; HCV, hepatitis C virus.

Kaplan–Meier analysis revealed a significantly higher cumulative incidence of HCC in patients with a score of ≥ 1 compared with that in patients with a score of 0 (log‐rank test, *p* < 0.001) (Figure 2). We prespecified a unit‐weighted composite (1 point each for Agile 3+ > 0.9398 and EOT‐AFP > 3.8 ng/mL) to favor simplicity and transportability. In a sensitivity analysis assigning 2 points to Agile 3+, discrimination and calibration were unchanged, so we retained unit weights as the primary model (Figure 3). The weighted specification (Agile 3+ = 2 points) yielded no meaningful improvement and no clear separation between 2‐ and 3‐point strata. Modeled as continuous variables, the C‐index was 0.784 (95% CI, 0.680–0.875) for the Agile 3–AFP score and 0.721 (95% CI, 0.582–0.852) for aMAP; the difference was not statistically significant (*p* = 0.570), indicating comparable discrimination.

### 2.4 Risk Factors for HCC Occurrence Following DAA Treatment

In the univariate analysis, male sex (HR 4.10, 95% CI 1.15–14.61, *p* = 0.03), liver cirrhosis (HR 7.63, 95% CI 2.60–22.36, *p* < 0.001), γ‐glutamyl transferase (GGT) at EOT (HR 1.01, 95% CI 1.00–1.01, *p* = 0.02), FIB‐4 index > 3.25 at EOT (HR 2.93, 95% CI 1.08–8.24, *p* = 0.04), and Agile 3‐AFP score ≥ 1 (HR 19.34, 95% CI 2.54–147.17, *p* = 0.004) were significantly associated with HCC development following DAA therapy (Table 4). Covariates (age ≥ 70 years, sex, liver cirrhosis, GGT, and FIB‐4) were selected based on prior reports of post‐SVR HCC risk and biological plausibility as core confounders [21, 22]. In the multivariate analysis, Agile 3‐AFP score ≥ 1 (HR 12.65, 95% CI 1.38–115.49, *p* = 0.02) and GGT (HR 1.00, 95% CI 1.00–1.01, *p* = 0.02) remained independent predictors of HCC occurrence, while liver cirrhosis and FIB‐4 index > 3.25 were no longer statistically significant (Table 4).

**TABLE 4:** Factors associated with HCC occurrence ≥ 1 year following HCV eradication using DAA treatment.

Factor	Univariate			Multivariate		
	HR	95% CI	*p*	HR	95% CI	*p*
Age ≥ 70 years	1.36	0.48–3.81	0.56			
Sex: male	4.10	1.15–14.61	0.03	3.33	0.89–12.43	0.07
LC	7.63	2.60–22.36	< 0.001	2.31	0.66–8.02	0.19
GGT (IU/L) at EOT	1.01	1.00–1.01	0.02	1.00	1.00–1.01	0.02
FIB‐4 index at EOT > 3.25	2.93	1.04–8.24	0.04	0.99	0.31–3.14	0.99
Agile 3+ and AFP score ≥ 1	19.34	2.54–147.17	0.004	12.65	1.38–115.49	0.02

*Note:* Data are expressed as hazard ratios (HRs) with 95% confidence intervals (CIs).

Abbreviations: CI, confidence interval; D HR, hazard ratio; DAA, direct‐acting antiviral; EOT, end of treatment; FIB‐4 index, fibrosis‐4 index; GGT, γ‐glutamyl transferase; HCC, hepatocellular carcinoma; HCV, hepatitis C virus; LC, liver cirrhosis; SOT, start of treatment.

### 2.5 Agile 3‐ AFP‐ GGT Scores

We constructed Agile 3‐AFP‐GGT score by adding 1 point for EOT‐GGT ≥ 67 IU/L (ROC‐derived cut‐off) to Agile 3‐AFP score. In analyses treating both predictors, the C‐index was 0.809 (95% CI 0.690–0.906) for Agile 3‐AFP‐GGT score and 0.784 (95% CI 0.680–0.875) for Agile 3‐AFP score. There was no statistical significance between both models (*p* = 0.740).

## 3 Discussion

In the present study, we demonstrated that a scoring model combining the baseline Agile 3+ score with serum AFP levels at EOT effectively stratified the risk of HCC in patients with chronic hepatitis C who achieved SVR following DAA therapy. Notably, patients with a total score of one or higher showed significantly increased HCC incidence, suggesting that this simple two‐factor model may be a practical tool for post‐SVR surveillance.

Previous studies have identified several risk factors for HCC after SVR, including advanced fibrosis, older age, male sex, and elevated AFP levels [23, 24, 25]. Our group previously reported that an EOT‐AFP level ≥ 3.8 ng/mL is associated with increased HCC risk [16]. However, LSM, a key noninvasive marker of fibrosis, was not included in that model [26, 27]. The Agile 3+ score incorporates LSM along with other clinical variables into a composite index and has shown robust diagnostic performance in MASLD and ALD populations [12]. There have been reports indicating that non‐alcoholic fatty liver disease and ALD are associated with an increased risk of HCC even after achieving SVR in patients with HCV infection [28, 29, 30].

To our knowledge, this is one of the first studies to evaluate the prognostic value of the Agile 3+ score for HCC development after DAA therapy in HCV‐infected patients. We found that an Agile 3+ score calculated before DAA initiation was significantly associated with subsequent HCC occurrence, with an optimal cutoff value of 0.9398. This threshold is notably higher than previously validated cutoffs for advanced fibrosis in MASLD populations, where scores > 0.675 define a high‐risk group [12].

Several factors may explain this discrepancy. First, the Agile 3+ score was initially developed and validated in nonviral liver diseases, particularly MASLD and ALD. Its application to HCV‐related liver disease has not been formally validated. Therefore, using the same cutoffs without recalibration may be inappropriate. Second, the score was calculated before antiviral treatment, a time when ALT and LSM are often elevated owing to ongoing inflammation. Because both parameters contribute to the Agile 3+ score, inflammatory activity may have led to overestimation of fibrosis severity. Third, the elevated Agile 3+ score may reflect not only fibrosis but also immune activation or hepatic injury factors that may play mechanistic roles in hepatocarcinogenesis. Thus, the score may represent a composite marker of both fibrotic and inflammatory burden, rather than fibrosis alone.

Taken together, our findings suggest that while the Agile 3+ score may be a useful predictor of HCC risk in HCV patients, its interpretation, particularly when calculated before treatment, should be approached with caution. Future prospective studies are needed to validate disease‐specific cutoffs and the optimal timing of score assessment.

Our results also emphasize that fibrosis‐related factors remain relevant even after viral eradication. Elevated LSM and metabolic risk factors such as DM may indicate persistent liver injury or latent carcinogenic potential. EOT‐AFP, in turn, may reflect biological activity associated with undetected dysplastic nodules or tumor‐promoting microenvironments. Combining structural (fibrosis) and functional (AFP) parameters likely improves risk prediction beyond either alone.

The FIB‐4 index, a well‐established noninvasive marker of liver fibrosis based on age, AST, alanine aminotransferase (ALT), and platelet count, has been extensively validated for predicting HCC in patients with chronic HCV infection. Several studies have reported that an FIB‐4 index ≥ 3.25 is associated with increased post‐SVR HCC risk [31, 32]. However, in our multivariate analysis including both the FIB‐4 index and the Agile 3+ with AFP score, only the Agile 3+ with AFP score remained as an independent predictor of HCC.

Clinically, this model offers a convenient and noninvasive approach to identify high‐risk individuals who may benefit from intensified HCC surveillance. Its binary scoring system (0–2 points) is easy to apply and relies solely on standard pre‐ and post‐treatment assessments. In the group with an Agile 3‐AFP score of 0, only one case of HCC was observed, suggesting that extending or even discontinuing surveillance may be considered. In this study, the Agile 3–AFP scores and aMAP scores showed comparable discrimination under continuous modeling. aMAP has the advantages of broad familiarity and routinely available variables with low missingness. Conversely, the Agile 3–AFP scores are mechanistically tailored to the HCV‐SVR context and may aid risk stratification in this population. In multivariable analysis, GGT emerged as an independent risk factor for HCC. However, adding GGT to the Agile 3–AFP score did not yield a statistically significant improvement in the C‐index; therefore, we adopted the simpler Agile 3–AFP score for the present study.

Several limitations should be acknowledged. First, this was a single‐center, retrospective study with a limited number of HCC cases. Validation in larger, multicenter cohorts is needed. Second, we were unable to incorporate post‐treatment LSM data, which may provide further prognostic insight regarding fibrosis regression. Ideally, the Agile 3+ score measured after SVR would offer a more accurate reflection of residual fibrotic burden and associated HCC risk. However, because of missing values, post‐treatment analysis was not feasible in our cohort. Third, the model assigns equal weight to both components. While this enhances simplicity, future studies should consider weighted scores to improve discriminative performance.

In conclusion, our study highlights the potential utility of a combined Agile 3+ score and EOT‐AFP score in predicting long‐term HCC risk after DAA therapy. This simple model may support risk‐adapted surveillance strategies in patients with SVR. Further research incorporating longitudinal post‐treatment data is warranted to optimize its clinical application.

## Consent

The authors have nothing to report.

## Conflicts of Interest

The authors declare no conflicts of interest.

## Data Availability

The data that support the findings of this study are available from the corresponding author upon reasonable request.
